# Genome-Wide Analysis of Biosynthetic Gene Cluster Reveals Correlated Gene Loss with Absence of Usnic Acid in Lichen-Forming Fungi

**DOI:** 10.1093/gbe/evaa189

**Published:** 2020-09-10

**Authors:** David Pizarro, Pradeep K Divakar, Felix Grewe, Ana Crespo, Francesco Dal Grande, Helge Thorsten Lumbsch

**Affiliations:** Departamento de Farmacología, Farmacognosia y Botánica, Facultad de Farmacia, Universidad Complutense de Madrid, Madrid 28040, Spain; Departamento de Farmacología, Farmacognosia y Botánica, Facultad de Farmacia, Universidad Complutense de Madrid, Madrid 28040, Spain; Department of Science & Education, The Field Museum, Chicago, Illinois; Departamento de Farmacología, Farmacognosia y Botánica, Facultad de Farmacia, Universidad Complutense de Madrid, Madrid 28040, Spain; Senckenberg Biodiversity and Climate Research Centre, Frankfurt am Main D-60325, Germany; LOEWE Center for Translational Biodiversity Genomics, Frankfurt am Main D-60325, Germany; Department of Science & Education, The Field Museum, Chicago, Illinois

**Keywords:** ascomycota, evolution, comparative-genomics, metabolic gene cluster, usnic acid, Parmeliaceae

## Abstract

Lichen-forming fungi are known to produce a large number of secondary metabolites. Some metabolites are deposited in the cortical layer of the lichen thallus where they exert important ecological functions, such as UV filtering. The fact that closely related lineages of lichen-forming fungi can differ in cortical chemistry suggests that natural product biosynthesis in lichens can evolve independent from phylogenetic constraints. Usnic acid is one of the major cortical pigments in lichens. Here we used a comparative genomic approach on 46 lichen-forming fungal species of the Lecanoromycetes to elucidate the biosynthetic gene content and evolution of the gene cluster putatively responsible for the biosynthesis of usnic acid. Whole-genome sequences were gathered from taxa belonging to different orders and families of Lecanoromycetes, where Parmeliaceae is the most well-represented taxon, and analyzed with a variety of genomic tools. The highest number of biosynthetic gene clusters was found in *Evernia prunastri*, *Pannoparmelia angustata*, and *Parmotrema austrosinense*, respectively, and lowest in *Canoparmelia nairobiensis*, *Bulbothrix sensibilis*, and *Hypotrachyna scytodes*. We found that all studied species producing usnic acid contain the putative usnic acid biosynthetic gene cluster, whereas the cluster was absent in all genomes of species lacking usnic acid. The absence of the gene cluster was supported by an additional unsuccessful search for ß-ketoacylsynthase, the most conserved domain of the gene cluster, in the genomes of species lacking usnic acid. The domain architecture of this PKS cluster—homologous to the already known usnic acid PKS cluster (*MPAS*) and CYT450 (*MPAO*)—varies within the studied species, whereas the gene arrangement is highly similar in closely related taxa. We hypothesize that the ancestor of these lichen-forming fungi contained the putative usnic acid producing PKS cluster and that the gene cluster was lost repeatedly during the evolution of these groups. Our study provides insight into the genomic adaptations to the evolutionary success of these lichen-forming fungal species and sets a baseline for further exploration of biosynthetic gene content and its evolutionary significance.

**Significance**

The fact that closely related lineages of lichen-forming fungi can differ in cortical chemistry suggests that natural product biosynthesis in lichens can evolve independent from phylogenetic constraints. Here we used a comparative genomic approach on 46 lichen-forming fungal species of the Lecanoromycetes to elucidate the biosynthetic gene content and evolution of the gene cluster putatively responsible for the biosynthesis of usnic acid. Whole-genome sequences were gathered and analyzed with a variety of genomic tools. All species lacking usnic acid production also lacked the putative biosynthetic gene cluster for usnic acid biosynthesis. This is the first study to report that the presence/absence of an entire biosynthetic gene cluster is putatively responsible for the biosynthesis of the corresponding secondary metabolite in lichen-forming fungi. Our results suggest that gene loss is an evolutionary mechanism underlying secondary metabolite diversity in lichens.

## Introduction

Lichens are fungi that form stable symbiotic relationships with algae and/or cyanobacteria (Nash 2008; Crespo et al. 2014). Lichen-forming fungi house in vegetative structures (lichen thalli) the photosynthetically active partner. Lichens are known to produce a large number of secondary metabolites (many of them extrolites) with almost 1000 known substances, the large majority of which exclusively found in lichen-forming fungi (Hawksworth 1976; Elix et al. 1984; Huneck and Yoshimura 1996; Lumbsch 1998; Stocker-Wörgötter 2008; Crespo et al. 2010; Calcott et al. 2018). Extrolites are deposited extracellularly, mostly in the medullary layer of the lichen thallus or in the cortical layer. Only a small number of substances occur in the cortex. Among those, coupled phenolics, which originate from polyketide pathways, such as depsides, depsidones, and usnic acids are found almost exclusively in lichens. In macrolichens, the most common cortical substances are the depsides atranorin or usnic acid. Usnic acids and cortical substances in general protect the photobiont from solar radiations as UV-B (reviewed in Solhaug and Gauslaa [2012]). The occurrence of these cortical substances is usually constant within clades (e.g., genera) and has been used to circumscribe genera (Lumbsch 1998) especially in Parmeliaceae, which is the largest family of lichen-forming fungi (Elix 1993; Crespo et al. 2010; Thell et al. 2012). This said cortical chemistry can be highly variable even among closely related genera, making the occurrence of these substances often scattered over the phylogenetic tree. This pattern suggests that natural product biosynthesis in lichens may evolve independently from phylogenetic constraints.

A previous ancestral character state reconstruction analysis suggested that the ancestor of parmelioid lichens, which is the largest group within Parmeliaceae, contained usnic acid and that this substance has been lost and replaced by atranorin several times independently (Divakar et al. 2013). However, the genetic mechanisms behind these repeated losses remained elusive.

Experimental work on lichen secondary metabolism is hampered by the fact that lichen-forming fungi are extremely slow growing. However, the recent applications of metagenomic approaches to the study of the lichen symbiosis can fortunately obviate the limitations imposed by the slow-growing nature of lichen-forming fungi and shed light on the underlying genetics of their secondary metabolism (Bertrand et al. 2018a). For example, a comparative genomic study identified a polyketide synthase (PKS) gene cluster putatively encoding for the biosynthesis of usnic acid (Abdel-Hameed et al. 2016). Genomic data sets are also increasing our knowledge of the presence and domain architecture of PKSs, which are key for the biosynthesis of fungal phenolics, at an unprecedented rate (Calchera et al. 2019).

The secondary metabolite biosynthetic pathways in filamentous fungi—including lichen-forming fungi—are typically organized into contiguous gene clusters in the genome, that is biosynthetic gene clusters (BGCs). These gene clusters contain the chemical backbone synthesis genes, such as nonribosomal peptide synthetases (NRPSs) and PKSs, tailoring enzymes, transporters and, often, transcription factors that control the expression of the clustered genes (Keller 2019). PKS catalyze repetitive condensations of an acetyl-coenzyme A (CoA) starter with malonyl-CoA units. Most of the fungal PKSs are type I PKSs, which consist of a set of ß-ketoacylsynthase (KS), acyl transferase (AT), and acyl carrier protein (ACP) domains. The PKSs are classified into non-reducing (NR−), partially reducing (PR−), and highly reducing (HR-PKS) types depending on the extent of chemical reduction in their polyketide structure (Miao et al. 1998; Bingle et al. 1999; Nicholson et al. 2001). Based on the phylogenetic relationships and domain architecture, the PKSs are further divided into subclades (Kroken et al. 2003). Within the NR-PKS eight groups (I to VIII) with known functions have been described in fungi so far (Liu et al. 2015). The genomes of ascomycetes fungi usually contain dozens of BGCs. These are either species specific or broadly taxonomically distributed and are often very different between species (Khaldi et al. 2010; Keller 2015). The total numbers of BGCs can differ widely even between very closely related species (Lind et al. 2015, 2017). Although the number and functions of BGCs have been largely studied in non-lichenized fungal model organisms, they are poorly known in lichen-forming fungal taxa (Calchera et al. 2019).

Here, we used a genomic approach to elucidate the biosynthetic gene content and the evolution of the PKS gene that putatively is central for the biosynthesis of usnic acid. We tested whether the presence and absence of usnic acid in a lichen is caused by differential expression of genes or reflects the presence of a gene cluster in the genome of a lichen-forming fungus. We specifically focused on the following research questions: 1) do all usnic acid-containing lichen-forming fungi share a homologous gene cluster?, 2) is this gene cluster present in lichen-forming fungi that do not produce usnic acid?, 3) is the gene cluster architecture conserved across Parmeliaceae? and 4) how can the presence or absence of the putatively usnic acid producing gene cluster be explained?

## Results and Discussion

### Genome Completeness to Detect Putative Usnic Acid BGC

Metagenomic methods recovered the large parts of the lichen-fungal genomes with a genome completeness varying from 70.51% to 98.4%. The lowest values of completeness were found for *Bulbothrix sensibilis* (70.5%) and *Usnea antarctica* (70.4%), and the highest in *Alectoria sarmentosa* (96.4%) and *Evernia prunastri* (95.3%). This heterogeneity allowed us to test whether the ability to detect the presence of the putative usnic acid gene cluster was dependent on the level of genome completeness. The fact that we detected the putative usnic acid cluster in *U. antarctica* demonstrates that the detectability of the targeted gene cluster and the level of genome completeness were independent in our study. The statistics of genome assemblies of the studied species are depicted in supplementary table S1, Supplementary Material online.

### BGC Content

**Fig. 1.**
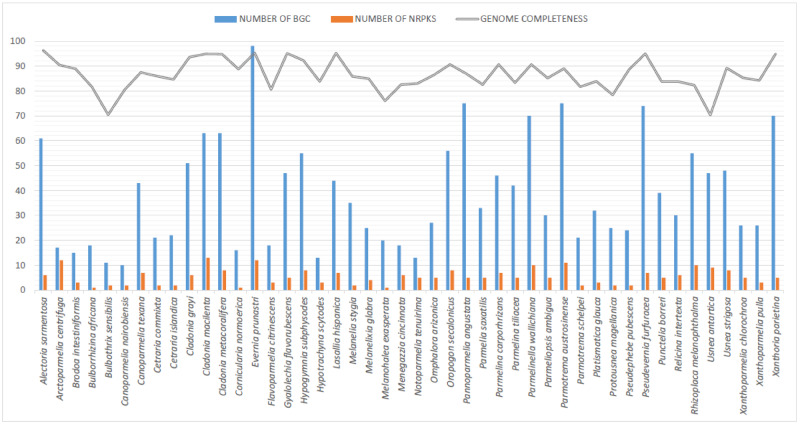
Genome completeness and BGC assessment of 46 lichen-forming fungi genomes used for this study. Bars represent the number of BGC (blue), number of non-reducing PKS (orange), and percentage of genome completeness (grey). The *Y*-axis represents the values of percentage and the number of BGCs.

The species with a higher number of BGCs were *E. prunastri* with 98 BGCs followed by *Pannoparmelia angustata* with 78 BGC and *Parmotrema austrosinense* with 75 BGCs. The species with lowest number were *Canoparmelia nairobiensis* with 10 BGCs, *B. sensibilis* with 11 BGCs, and *Hypotrachyna scytodes* with 13 BGCs (fig. 1). Although the BGCs have been studied for individual species in lichen-forming fungi (see e.g. Armaleo et al. 2011; Bertrand et al. 2018b; Dal Grande et al. 2018; Calchera et al. 2019), this has not yet been compared at large scale for example family level. To our knowledge, this is the first study comparing BGC contents in species belonging to different major clades of the family Parmeliaceae (fig. 1). Within Parmeliaceae, 80 BGCs have been reported in *E. prunastri* and 51 in *Pseudevernia furfuracea* (Calchera et al. 2019); in Cladoniaceae, *Cladonia uncialis* contained 48 BGCs (Bertrand et al. 2018b); in Teloschistaceae, *Caloplaca flavorubescens* had 13 BGCs (Park et al. 2013); and in Umbilicariaceae, *Lasallia hispanica* contained 18 BGCs (Dal Grande et al. 2018). Although the highest number of BGCs has been found in some Parmeliaceae taxa (fig. 1), no correlations between BGCs and secondary metabolism or ecology were found. For example, *E. prunastri* with a high number of BGCs (98) contained three main secondary metabolites (atranorin, usnic, and evernic acids) whereas *P. furfuracea* with a relatively low number of BGCs (51) also contained a similar number of main substances (atranorin, physodic, and olivetoric acids) (supplementary table S2, Supplementary Material online). Both species are found in same ecological conditions and widespread in temperate habitats*. Parmotrema austrosinense* with a high number of BGCs (75) contained two main substances (atranorin and lecanoric acid) (supplementary table S2, Supplementary Material online) and are widely distributed in the tropical habitats. Nonetheless, a genome-wide examination of BGC contents provides a road map to the genomic changes essential for the extensive diversity of secondary metabolites in this group of lichen-forming fungi. Parmeliaceae is one of the most diverse families both in species diversity and in containing a large number of different secondary metabolites. Usnic acid and atranorin are the most common cortical substances in the family. Usnic acid protects the photobiont from solar radiations as UV-B and act as defense against herbivores (Solhaug and Gauslaa 2012) and has been attributed to adaptive radiation in Parmeliaceae occupying different climatic regions, for example *Xanthoparmelia* in semi-arid regions (Lumbsch et al. 2008; Divakar et al. 2013). This is the most diverse genus of lichen-forming with more than 800 described species; of these over 600 species contained usnic acid.

### Phylogeny of Non-Reducing PKS

**Fig. 2.**
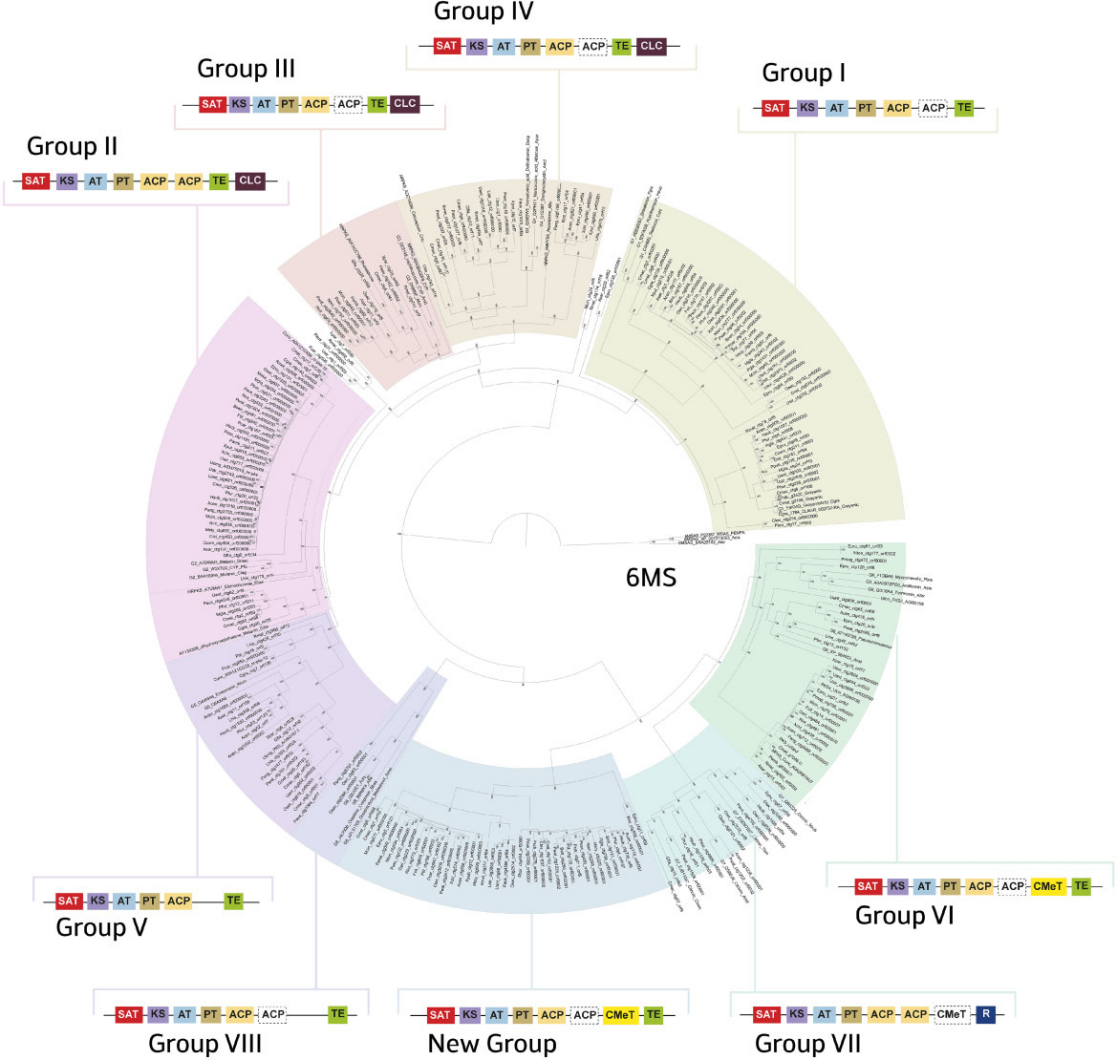
Gene tree of NR-PKS dataset inferred by ML analysis in IQ-Tree using 6 Methylsalicylic PKS protein sequences as outgroup. The distinct colors represent the different groups corresponding to the domain arrangement. In each group the domain arrangement of NR-PKS is highlighted with distinct colors. SAT = starter unit-ACP transacylase, KS = ketosynthase, AT = acyl transferase, PT = product template, ACP = acyl carrier protein, TE = thioesterase, TE/CLC = thioesterase/Claisen cyclase, CMeT = C-methyltransferase, R = reductase.

A NJ phylogeny of the KS domain and full PKS sequence revealed the evolutionary relationship of biosynthetic gene content and the origins of the putative usnic acid PKS genes in lichen-forming fungi. The KS domain and PKS full-length sequence showed highly similar tree topology (supplementary fig. S2, Supplementary Material online) and therefore only PKS phylogeny is discussed in detail. The data matrix of amino acid sequences of PKS domain consists of 356 columns (residues) and 167 taxa (of these 45 were from curated reference data). All sequences included in the analysis passed the composition chi^2^-test (*P* < 5%) and the best substitution model was LG+I+G4 according to BIC. The phylogenetic tree inferred from the PKS data matrix was rooted using polyketide 6-methylsalicylic acid sequences. In the resulted tree, two main groups were found among NR-PKSes (fig. 2). The well-supported topology is concordant with previous reported KS phylogenies (Liu et al. 2015; see Supplementary fig. S1, Supplementary Material online).

The first monophyletic cluster comprised the groups I–V of NR-PKS. The group I included PKS sequences for the biosynthesis of aromatic compound derived from orsellinic acid, such as grayanic acid in *Cladonia grayi* (Armaleo et al. 2011) or mycotoxins, such as zearalenone in *Fusarium gramineanum* (Gaffoor and Trail 2006). The PKS of this group shows a domain arrangement of SAT, KS, AT, PT, one or two ACP domains and TE. Group V comprised PKS without the TE domain and is implicated in the production of different mycotoxin, such as desertorin (*Aspergillus nidulans*) or atrochrysone (*Aspergillus fumigatus*) (Galagan et al. 2005; Lim et al. 2012). Groups II–III–IV formed a supported sister-relationship to group V, comprising PKS with TE/CLC domain located on the N-terminal with a similarity in the domain arrangement. Group II contained PKS involved in melanin biosynthesis as PKS1 of *Exophiala dermatiditis* (Feng et al. 2001) and it is characterized by having two ACP domain between PT and TE; in this group, many homologous genes to melanin biosynthesis of some Pezizomycotina fungi were found in lichen-forming fungi. This could indicate that the NR-PKS group II could be responsible for the biosynthesis of melanin in lichen-forming fungi. Moreover, homology search using a known gene clusters have been applied for the identification of related gene clusters in other fungal genomes (Gardiner and Howlett 2005; Khaldi et al. 2010). However, an additional study will be necessary in order to confirm its function in lichens. Groups III and IV NR-PKS contain proteins that synthesize large polyketides chains as conidial yellow pigment Alb1 (group III) or aflatoxin/sterigmatocystin of *A. nidulans* (Yu and Leonard 1995) (group IV) (supplementary table S3, Supplementary Material online).

The second monophyletic cluster included the groups VI–VII–VIII and an undescribed group (named hereafter “New Group”) sister to VI and VII group, which does not contain PKS of known function. As eight NR-PKS groups with known functions have been described in fungi (Liu et al. 2015), our results suggest that this group with unknown function may belong to a new NR-PKS group. This result can serve as valuable entry point to search for functional gene clusters in fungi. However, we refrain to describe this group formally as this is not the main focus of our study and an additional study will be needed. Sequences located on the groups VI, VII and the New Group are largely characterized by having a methylation domain (CMet) between the ACP domain and the N-terminal domain. Group VII, which is responsible for the biosynthesis of citrinin in many fungal species (*A. nidulans, Coccidioides immitis*, supplementary table S3, Supplementary Material online) (Chiang et al. 2009; Gallo et al. 2013), is characterized by having a reductase (R) domain placed on the N-terminal. The domain arrangement of this probable new group is similar to group VI. Although the new group included only lichen-forming fungi, the blast analysis found homologous sequences belonging to other groups of fungi with unknown functions. The PKS putatively responsible for usnic acid biosynthesis in lichens was found in NR-PKS group VI (fig. 2). We found that all producer species contained PKS sequences homologous to the putative PKS of usnic acid of *C. uncialis (MPAS)* (Abdel-Hameed et al. 2016). This orthologous gene cluster was absent in all non-producer species.

**Fig. 3.**
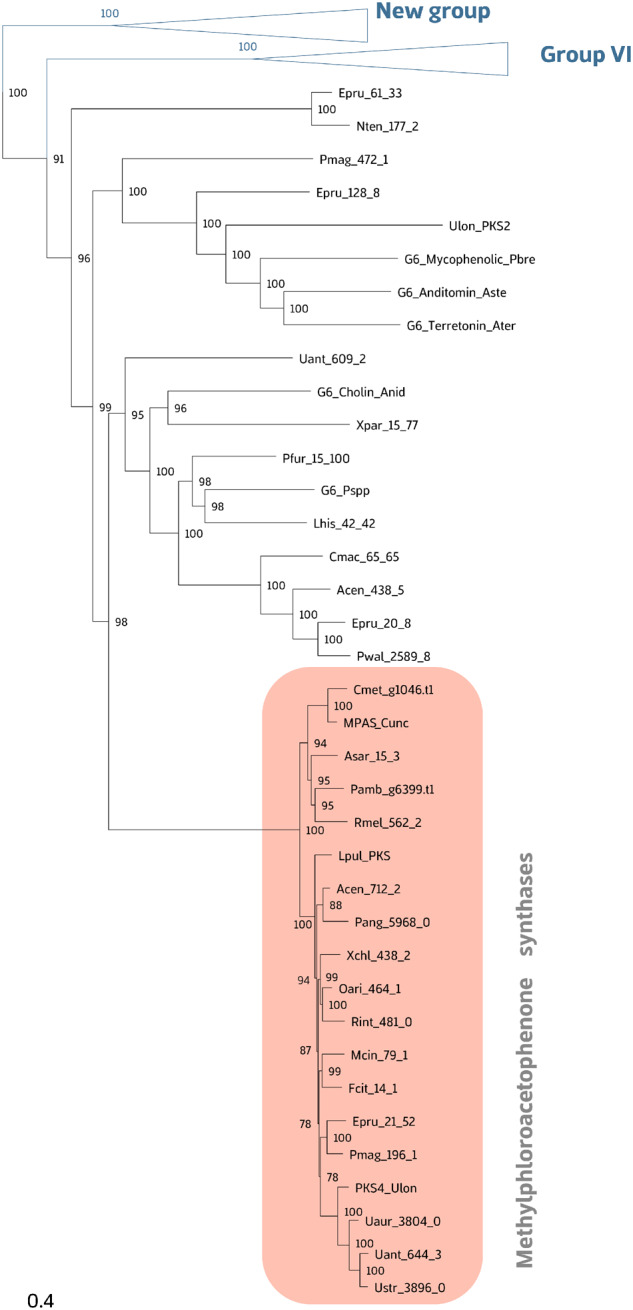
Fragment of NR-PKS tree showing the relationship of putative PKS responsible for the biosynthesis of usnic acid.

Within group VI, a strong phylogenetic structure was observed. Three main strongly supported (>95 bootstrap) monophyletic groups were found, in accordance with the domain arrangement of the cluster. The short phylogenetic branches of usnic acid PKS gene cluster and the concordance in phylogenetic relationships between PKS phylogeny and species phylogeny (see, e.g., *Usnea*, fig. 3) (Pizarro et al. 2018) indicate that the scattered occurrence of usnic acid across the phylogenetic tree of these lichen-forming fungi may not be the result of horizontal gene transfer (Kroken et al. 2003; Bushley and Turgeon 2010; Campbell et al. 2013; Lind et al. 2017).

### Gene Arrangement of Putative Usnic Acid BGC

**Fig. 4.**
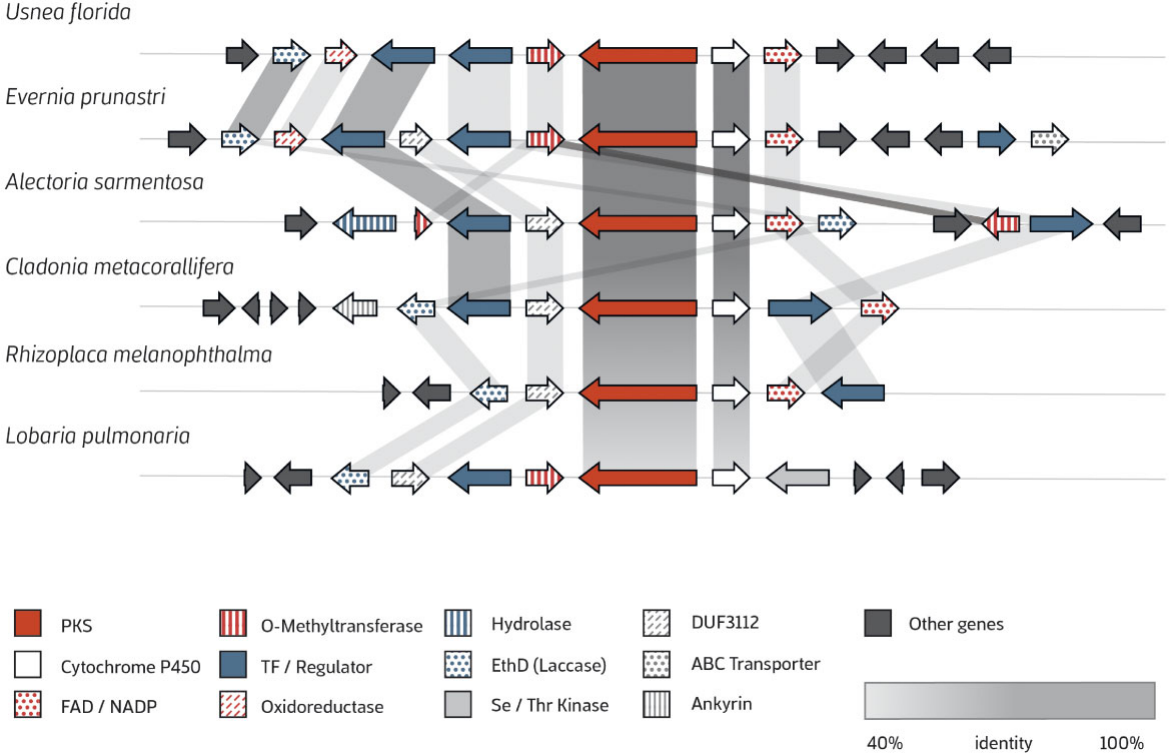
Putative usnic acid compound cluster (UA) conservation and synteny. The UA cluster from other lichen-forming fungi resembled to the characterized usnic acid core genes PKS (MPAS) and Cytochrome P450 (MPAO) from *C. uncialis* (high percentage of identity in protein-by-protein comparisons of both genes in all species included).

We then compared gene cluster architecture of the putative usnic acid BGC among six species of Lecanoromycetes. All genomes of producer species shared homologous genes, that is a core NR-PKS biosynthetic gene (corresponding to *MPAS* in *C. uncialis*), a cytochrome P450 gene (*CYT450*) (corresponding to *MPAO* in *C. uncialis*) (Abdel-Hameed et al. 2016) and one or two putative transcription factors (fig. 4). The putative transcription factors (in red in fig. 4) contain Cys6-Zn domains, as often found in regulators of the secondary metabolism in fungi (Shelest 2017). The gene arrangement of the BGC in *Usnea florida* is very similar to those of the other Parmeliaceae species, *E. prunastri* and *A. sarmentosa*. They share homologous genes that could be likely involved in the usnic acid biosynthesis like the Lacasse (Tsai et al. 1999) and the putative oxidoreductase gene, which could be implicated in the oxidation of precursors molecules of usnic acid.

We found an O-methyl transferase gene flanking the core biosynthetic PKS genes in all species, except *Cladonia metacorallifera* and *Rhizoplaca melanophthalma*. This O-methyltransferase gene is common to many BGC and is homologous to the coactivator *AflJ*, implicated in the biosynthesis of aflatoxin in *Aspergillus parasiticus* (Chang 2003) and to *mdpA* in *A. nidulans* (Chiang et al. 2009). In the 5ʹ flanking upstream region of the *CYT450*, we found a FAD/NADP-containing protein in all species included with exception of *Lobaria pulmonaria*, which contained a serin/threonin kinase. *Evernia prunastri*, *A. sarmentosa*, *C. metacorallifera*, and *R. melanophthalma* contained a gene DUF3112 with unknown function, which was in different locations depending on the species. For example, in *E. prunastri* it is located between the regulators, whereas in *A. sarmentosa* it was between the PKS gene and a regulator. An ABC transporter that may be implicated in the secretion of usnic acid was only found in the *E. prunastri*.

### Repeated Independent Usnic Acid BGC Losses in Non-Producers

**Fig. 5.**
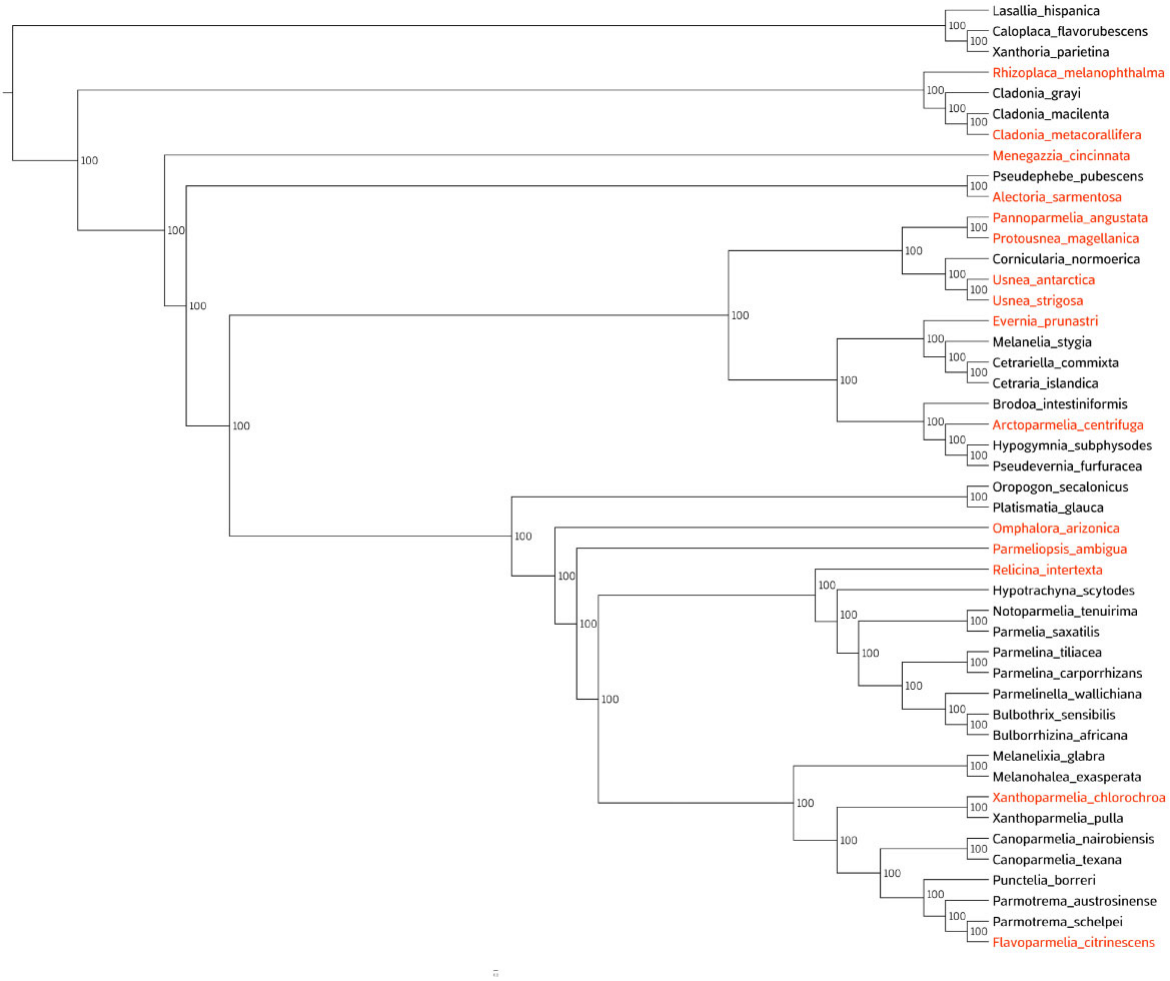
Phylogenetic tree from the IQ-Tree analysis based on a concatenated dataset of 2556 BUSCO genes (Pizarro et al. 2018). Numbers at the nodes represent ML bootstrap support values based on 1,000 bootstrap pseudoreplicates. Species in orange and black indicate the usnic acid producer species and non-producer species, respectively.

The phylogenetic tree inferred from the concatenated dataset of 2556 BUSCO genes showed similar results as in our previous study (fig. 5; Pizarro et al. 2018). All nodes in the tree were maximally supported (100%). Usnic acid-producing species were widely spread across the Lecanorales. All families included in this study had representatives of usnic acid-producing species, such as *R. melanophthalma* (Lecanoraceae), *C. metacorallifera* (Cladoniaceae), and 13 usnic acid-producing species representing the 6 major clades of the Parmeliaceae (see fig.5). The orthologous putative usnic acid BGC was absent in all studied species lacking usnic acid whereas it was present in all studied usnic acid producer species (fig. 5). Interestingly, in some cases producer and non-producer species formed strongly supported sister-group relationship, for example *Cladonia macilenta* (non-producer) and *C. metacorallifera* (producer), *Xanthoparmelia pulla* (non-producer) and *Xanthoparmelia chlorochroa* (producer). This strongly suggests that the entire usnic acid BGC was lost independently several times during the evolution of this fungal group. Similar results have been found in other groups of BGC and fungi, for example the bikaverin BGC was entirely lost in different *Botrytis* species (Campbell et al. 2013).

The discontinuous distribution of PKS genes among fungal groups has been often explained by gene loss and convergent evolution (Stayton 2015) rather than horizontal gene acquisition (see, e.g. Kroken et al. 2003; Bushley and Turgeon 2010; Campbell et al. 2012; Lind et al. 2017). Corroborating this scenario of repeated gene cluster losses, the amino acid sequences of the putative usnic acid biosynthetic genes were highly similar and showed similar tree topology as the species tree (see figs. 3 and 5). Furthermore, within the putative usnic acid PKS gene phylogeny, the branches were homogenous and short, and no long branches were detected (see fig. 3). In the 2,556-gene phylogeny, the species containing the usnic acid BGC (i.e. producer) do not form one monophyletic cluster. Instead, producers and non-producers were part of a large monophyletic group, suggesting an ancestral state of studied gene cluster in this fungal group (Lind et al. 2017). This is in accordance with the previous finding supporting an ancestral state of usnic acid production in the parmelioid lichens, the major clade within Parmeliaceae (Divakar et al. 2013). Due to limited taxon sampling, a more conclusive ancestral state reconstruction analysis was not possible, and thus remains a hypothesis to test in future studies. On the other hand, the secondary acquisition or horizontal gene transfer may be possible but it is less probable in our case as. The evolutionary relationships among Lecanorales taxa were highly similar to those published previously (Pizarro et al. 2018) and therefore are not discussed in detail here.

## Conclusions

Here we present a first comprehensive study investigating biosynthetic gene contents in the genomes of the largest clade of lichen-forming fungi. A total of eight NR-PKS groups (I–VIII) with known functions and, in addition, one novel NR-PKS group with unknown function in fungi were identified. In this study, we showed that, all species lacking usnic acid production also lacked the putative BGC for usnic acid biosynthesis. To our best knowledge, this is the first study to report that the presence/absence of an entire BGC is putatively responsible for the biosynthesis of the corresponding secondary metabolite in lichen-forming fungi. The short phylogenetic branches of usnic acid PKS gene cluster, the concordance in phylogenetic relationships between PKS phylogeny and species phylogeny and high similarity in the amino acid sequences of this BGC among all usnic acid producer species, suggest that the discontinuous distribution of usnic acid across the phylogenetic tree of these lichen-forming fungi may not be explained by horizontal gene transfer. Rather, we hypothesized that the usnic acid BGC might have been entirely lost in the non-producer species during the evolution of these fungi. Our results suggest that gene loss is an evolutionary mechanism underlying secondary metabolite diversity in lichens. Furthermore, our study sets the path for future research on the molecular detection of producer species and the metabolic engineering of lichen secondary metabolite biosynthesis. Investigating gene losses has great potential to reveal the association of BCG with natural compounds and to understand the genomic basis underlying ecological and metabolic changes.

## Materials and Methods

### Taxon Sampling

A total of 46 lichen-forming fungal species of producers and non-producers of usnic acid were included in this study. We included genomes of species belonging to different orders of Lecanoromycetes including Teloschistales (*Xanthoria parietina* and *Gyalolechia flavorubescens*), Umbilicariales (*Umbilicaria hispanica*; Dal Grande et al. 2018), and Lecanorales (family Cladoniaceae: *C. grayi, C. macilenta, C. metacorallifera*; Lecanoraceae: *R. melanophthalma*; Parmeliaceae: 39 species representing six of its seven major clades; see supplementary table S2, Supplementary Material online).

### Genome Sequencing and Assembly

Total genomic DNA of 38 specimens of Parmeliaceae was extracted from apothecia or thalli using the Quick-DNA™ Fungal/Bacterial Miniprep Kit (Zymo Research, Irvine, CA) following the manufacturers’ instructions. Genome sequencing, assembly, and taxonomic assignment were carried out as described in Pizarro et al. (2018).

### Genome Completeness Assessment and Phylogenomic Analysis

BUSCO analysis was used to evaluate the genome completeness (Simão et al. 2015). Here, we used the Pezizomycotina data set containing 3,156 single-copy genes to assess the completeness of the 46 genomes of this study. To infer the phylogenetic relationships between producers and non-producers of usnic acid, every genome was explored following a methodology as described before (Pizarro et al. 2018). The complete BUSCO single-copy genes predicted in each genome were extracted and aligned using MAFFT L-INS-i (Standley 2013). A supermatrix was created by concatenating all alignments using FASconCAT.pl (Kück and Longo 2014). Evolutionary relationships were inferred from this subset using maximum likelihood (ML) analysis implemented in IQTree v1.5.5 (Nguyen et al. 2015; Kalyaanamoorthy et al. 2017) with standard model selection and 1,000 bootstrap replicates. The resulting tree was visualized using FigTree 1.3.1 (Rambaut 2009).

### BGC Prediction and Usnic Acid Genes Cluster Identification

In order to identify the putative usnic acid PKS cluster, gene prediction and annotation were performed in every genome using AUGUSTUS (Stanke et al. 2004) and MAKER2 (Holt and Yandell 2011). In parallel, we used every genome and its respective gene prediction as input for antiSMASH 4.0 (Blin et al. 2017) for secondary metabolites gene cluster prediction. Afterwards, MPAS of *C. uncialis* (A0A0R8YWJ7) and the orthologous gene PKS4 of *Usnea longissima* (AGI60156) sequences were downloaded from Uniprot database and HMM profile was created using MAFFT and HMMER hmmbuild (Mistry et al. 2013). These profiles and hmmsearch tool were used to search the most similar sequences present in every protein data set predicted from each genome. Only non-reducing PKSes were considered for further analyses.

### Full-Length NR-PKS and KS Domain Phylogenetic Analysis

We used Hidden Markov Model profiles for the identification of a specific type of domain. The recovered NR-PKS proteins were scanned using HMMER against the Pfam (Mitchell et al. 2016) protein domain collection. Then using a bioinformatic approach with BEDtool (getfasta) (Quinlan and Hall 2010), the different KS domains from PKS were identified in the corresponding amino acid sequences. An identical procedure was carried out over an additional curated NR-PKS data set (see supplementary table S3, Supplementary Material online) of characterized fungal NR-PKSs. Multiple alignments were performed using MAFFT L-INS-I over the amino acid sequence of full-length PKS dataset and KS domain dataset. Evolutionary relationships were inferred using ML analysis implemented in IQ-Tree v1.5.5 with standard model selection. For each analysis, a Neighbour Joining and 1,000 bootstrap replicates were calculated using fast bootstrapping option. The resulting phylogenetic trees were rooted with 6 Metylsalicylic PKS (6MS) sequences and drawn using FigTree v1.3.

### Synteny Comparison of Usnic Acid BGC

Genomes that contain the full putative gene clusters of usnic acid biosynthesis were selected for synteny comparison. Since some of the usnic acid producer species had low gene cluster completeness, only *C. macilenta, A. sarmentosa*, *E. prunastri*, and *R. melanophthalma* were considered for the analysis. In addition, *U. florida* and *L. pulmonaria*, downloaded from JGI, were also included in the analysis. Using Orthofinder (Emms and Kelly 2015), we initially compared the homology among different genomes and subsequently drew their relationship.

## Supplementary Material

Supplementary material is available at *Genome Biology and Evolution* online (http://www.gbe.oxfordjournals.org/).

## Supplementary Material

evaa189_Supplementary_Data
