# Supplementary material for: Genome-Wide Analysis of Biosynthetic Gene Cluster Reveals Correlated Gene Loss with Absence of Usnic Acid in Lichen-Forming Fungi
Source: Genome Biol Evol. 2020 Sep 10;12(10):1858–68. doi: 10.1093/gbe/evaa189 (PMC7643366; doi:10.1093/gbe/evaa189)
Supplement: evaa189_Supplementary_Data [file evaa189_supplementary_data.pdf]

Supplementary Materials

Supplementary Fig. S1. Gene tree of KS domain dataset inferred by ML analysis in IQtree using 6MS protein sequence as outgroup.

Supplementary Table S1. Statistics of the genome assemblies of species used in this study.

Supplementary Table S2. List of species included in the analysis with number of predicted BGC and secondary metabolites recovered from literature.

Supplementary Table S3. List of additional sequences included on the PKS gene phylogeny with their corresponding ProteinID, metabolic product when is known and the subgroup of NR-PKS.

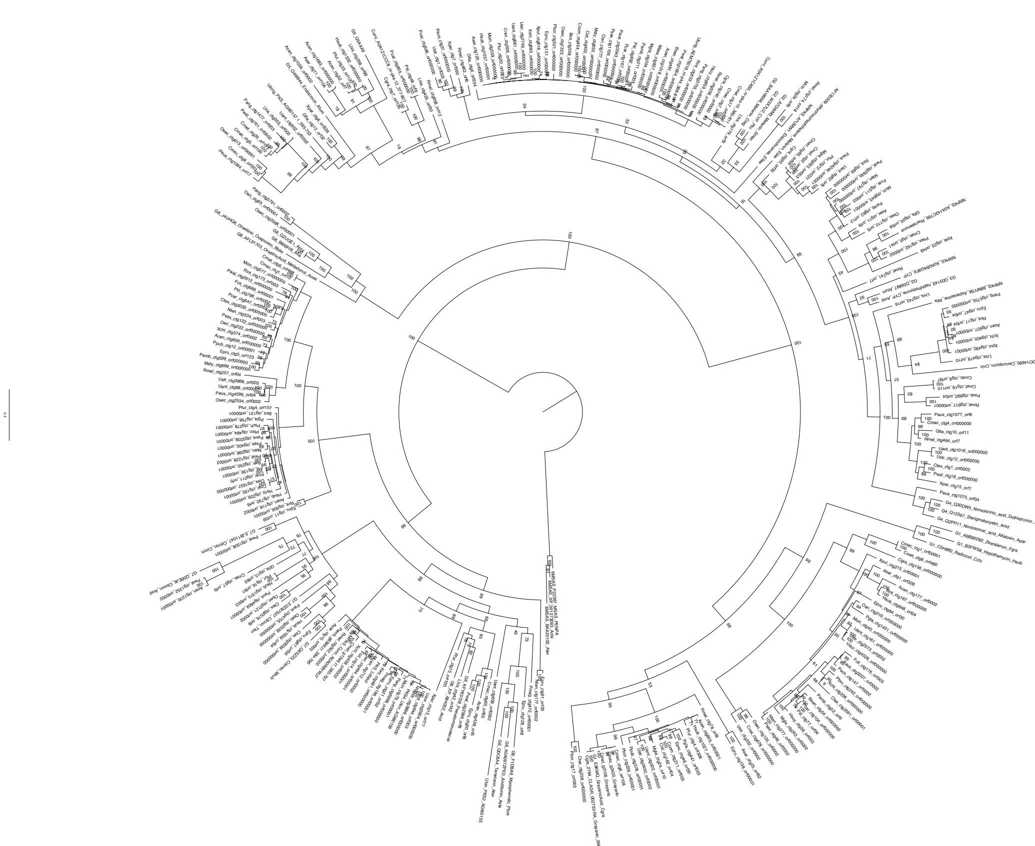

Species	Total Length	GC Content	Total Contigs	N50	Depth
Alectoria sarmentosa	33052253	48.35	3342	25327	53.9766
Arctoparmelia centrifuga	40459659	47.29	6931	23666	74.2259
Brodoa intestiniformis	38270146	47.46	11324	16037	23.6498
Bulborrhizina africana	30662347	47.95	3502	17258	32.3507
Bulbothrix sensibilis	26829917	48.68	5085	9396	9.81746
Canoparmelia nairobiensis	27201108	49.27	4073	11657	18.6761
Canoparmelia texana	36952011	49.03	10351	16147	75.2697
Cetraria commixta	33545404	48.11	7219	15855	30.6885
Cetraria islandica	51316390	44.41	3472	95207	32.8847
Cladonia grayi	34622149	44.44	414	243412	-
Cladonia macilenta	36862809	44.77	66	1469036	-
Cladonia metacoralifera	36682060	44.91	30	1591850	-
Cornicularia normoerica	30301608	47.72	13048	19303	32.3367
Evernia prunastri	40346456	48.97	227	264454	-
Flavoparmelia citrinescens	26342035	49.24	4828	12180	17.7841
Gyalolechia flavorubescens	34468235	41.89	36	1693300	-
Hypogymnia subphysodes	36702972	49.66	31631	36657	95.3139
Hypotrachyna scytodes	28847286	49	17991	14076	23.6407
Lasallia hispanica	41207996	51.28	1619	145035	-
Melanelia stygia	31606081	49.12	6915	21268	18.313
Melanelixia glabra	29221822	49.84	3214	17361	23.8734
Melanohalea exasperata	29551578	49.77	10174	8923	28.9029
Menegazzia cincinnata	32469692	49.12	26921	79423	12.8519
Notoparmelia tenuirima	29178179	47.87	4734	14279	16.5765
Omphalora arizonica	28699306	48.76	3711	16491	43.1317
Oropogon secalonicus	37000476	56.09	194774	14638	87.011
Pannoparmelia angustata	47561874	52.42	60516	9925	17.0085
Parmelia saxatilis	30564937	49.62	5762	16605	23.0036
Parmelina carporhrizans	34751387	48.78	11765	9802	-
Parmelina tiliacea	31229044	49.04	3579	17640	29.131
Parmelinella wallichiana	36254821	48.76	22351	17440	74.3405
Parmeliopsis ambigua	32514193	48.27	6866	21266	28.1013
Parmotrema austrosinense	43516517	49.46	9175	14288	73.5369
Parmotrema schelpei	29016443	48.51	7361	13723	14.5869
Platismatica glauca	28681065	51.1	3607	17398	19.534
Protousnea magellanica	30922013	48.5	6278	11553	19.921
Pseudephebe pubescens	29125554	48.72	3646	19539	50.068
Pseudevernia furfuracea	37795223	47.86	46	1178799	-
Punctelia borreri	31384725	48.42	4528	15250	83.8563
Relicina intertexta	31836904	47.79	15979	12463	37.048
Rhizoplaca melanophthalma	33087415	48.8	1430	49682	-
Usnea antartica	24119594	48.79	1100	23562	2.12917
Usnea strigosa	37557709	48.92	8736	14477	65.1026
Xanthoparmelia chlorochroa	28326562	49.27	3078	18494	26.1129
Xanthoparmelia pulla	27802724	49.11	7345	14590	24.0985
Xanthoria parietina	31900637	49.89	39	1731186	-

Species	Synonym	Usnic Acid	Number of Metabolic
Alectoria sarmentosa	Asar	Producer	61
Arctoparmelia centrifuga	Acen	Producer	17
Brodoa intestiniformis	Bint	Non-producer	15
Bulborrhizina africana	Bafr	Non-producer	18
Bulbothrix sensibilis	Bsen	Non-producer	11
Canoparmelia nairobiensis	Cnai	Non-producer	10
Canoparmelia texana	Ctex	Non-producer	43
Cetraria commixta	Ccom	Non-producer	21
Cetraria islandica	Cisl	Non-producer	22
Cladonia grayi	Cgra	Non-producer	51
Cladonia macilenta	Cmac	Non-producer	63
Cladonia metacoralifera	Cmet	Producer	63
Cornicularia normoerica	Cnor	Non-producer	16
Evernia prunastri	Epru	Producer	98
Flavoparmelia citrinescens	Fcit	Producer	18
Gyalolechia flavorubescens	Gfla	Non-producer	47
Hypogymnia subphysodes	Hsub	Non-producer	55
Hypotrachyna scytodes	Hscy	Non-producer	13
Lasallia hispanica	Lhis	Non-producer	44
Melanelia stygia	Msty	Non-producer	35
Melanelixia glabra	Mgla	Non-producer	25
Melanohalea exasperata	Mexa	Non-producer	20
Menegazzia cincinnata	Mcin	Producer	18
Notoparmelia tenuirima	Nten	Non-producer	13
Omphalora arizonica	Oari	Producer	27
Oropogon secalonicus	Osec	Non-producer	56
Pannoparmelia angustata	Pang	Producer	75
Parmelia saxatilis	Psax	Non-producer	33
Parmelina carporhizans	Pcar	Non-producer	46
Parmelina tiliacea	Ptil	Non-producer	42
Parmelinella wallichiana	Pwal	Non-producer	70
Parmeliopsis ambigua	Pamb	Producer	30
Parmotrema austrosinense	Paus	Non-producer	75
Parmotrema schelpei	Psch	Non-producer	21
Platismatica glauca	Pgla	Non-producer	32
Protousnea magellanica	Pmag	Producer	25
Pseudephebe pubescens	Ppub	Non-producer	24
Pseudevernia furfuracea	Pfur	Non-producer	74
Punctelia borreri	Pbor	Non-producer	39
Relicina intertexta	Rint	Producer	30
Rhizoplaca melanophthalma	Rmel	Producer	55
Usnea antartica	Uant	Producer	47
Usnea strigosa	Ustr	Producer	48
Xanthoparmelia chlorochroa	Xchl	Producer	26
Xanthoparmelia pulla	Xpul	Non-producer	26
Xanthoria parietina	Xpar	Non-producer	70

Compounds
Usnic acid, alectoronic acid, olivetoric acid
Usnic acid, alectoronic acid, protocetraric acid
Atranorin, prcinol, B-orcinol depsidons
Atranorin, gyrophoric acid, lecanoric acid
Atranorin, gyrophoric acid, lecanoric acid
Atranorin, chloroatranorin, Divaricatic/nordivaricatic acid
Atranorin, divariatic acid,stictic acid
A-collatolic acid
Fumarprotocetraric acid, protolichesterinic, lichesterinic acid
Grayanic acid, protocetraric acid, fumarprotocetraric acid, divaronic acid, stenophoric acid
Barbatic, squamatic,thamnolic, didymic acid (some Usnic acid)
Usnic acid, didymic acid, squamatic acid, barbatic acid
No compounds
Atranorin, Usnic acid, Evernic acid
Usnic acid, Atranorin, protocetraric acid, caperatic acid
Antraquinon
Atranorin, physodic acid, protocetraric acid, physodalic acid
4-O-demethylbarbatic acid, atranorin, barbatic acid, chloroatranorin, echinocarpic acid
Gyrophoric acid, papulosin, antraquinon
Fumarprotocetraric acid, caperatic acid
Lecanoric acid
Fumarprotocetraric acid, norstictic acid
Atranorin, depsides and depsidones
Atranorin, chloratranorin; lobaric acid, salazinic, consalazinic, protocetra-ric and fumarprotocetraric
Usnic acid
Secalonic Acid
Usnic acid, divaricatic acid
Atranorin, salazilic acid, lobaric and norstictic acid)
Atranorin, lecanoric acid
Atranorin, lecanoric acid
Atranorin, salazilic acid
Usnic acid, atranorin, divaricatic acid
Atranorin, lecanoric acid
Stictic acid, norstictic acid, protocetraric acid
Atranorin, caperatic acid
Usnic acid, sekikaic acid
No compounds
Atranorin, physodic acid, olivetoric acid, lecanoric acid
Atranorin, gyrophoric acid
Usnic acid, protocetraric acid
Usnic acid, psoromic acid, placodiolic acid, lecanoric acid, psoromic acid
Usnic acid, barbatic acid, diffractaic acid, squamatic acid, salazinic acid, norstictic acid, stictic acid
Usnic acid, barbatic acid, diffractaic acid, squamatic acid, salazinic acid, norstictic acid, stictic acid
Usnic acid, salazinic acid, norstictic acid
Gyrophoric acid, stenophoric acid, divaricatic acid
Parietin

acids, protolichesterinic acid, echinocarpicacid acid

Specie	**Abbreviation**	**Protein ID**
Armillaria mellea	Amel	AFL91703
Aspergillus clavatus	Acla	XP_001273093
Aspergillus flavus	Afla	B8MYS6
Aspergillus flavus	Afla	B8N9Y8
Aspergillus oryzae	Apar	Q2PH11
Aspergillus oryzae	Aory	Q2UGE1
Aspergillus terreus	Ater	Q0C8A4
Aspergillus terreus	Ater	BAA20102
Beauveria bassiana	Bbas	J4UHQ6
Chaetomium chiversii	Cchi	C5H882
Cladonia grayi	Cgra	E9KMQ
Cladonia uncialis subsp. uncialis	Cunc	A0A1Z1C4B5
Cladonia uncialis subsp. Uncialis	Cunc	A0A1Z1CCC8
Cladonia uncialis subsp. Uncialis	Cunc	A0A0R8YWJ7
Coccidioides immitis	Cimm	EJB11047
Dothistroma septosporum	Dsep	Q30DW5
Elsinoe fawcettii	Efaw	A7UMW1
Emericella nidulans	Anid	Q03149
Emericella nidulans	Anid	Q12397
Emericella nidulans	Anid	Q5AXA9
Emericella nidulans	Anid	XP_664052
Emericella nidulans	Anid	Q5BEJ6
Emericella variicolor	Aste	A0A097ZPE0
Exophiala dermatitidis	Eder	AF130309
Fusarium gramineanum	Fgra	ABB90282
Glomerella lagenarium	Clag	BAA18956
Hypomyces subiculosus	Hsub	B3FWS8
Lobaria pulmonaria	Lpul	PKS_Lobpul
Metarhizium guizhouense	Mgui	A0A0B4G9F9
Monascus purpureus	Mrub	Q65Z23
Mycosphaerella coffeicola	Cnic	ADO14690
Neosartorya fumigata	Afum	O59897
Neosartorya fumigata	Afum	Q4W944
Penicillium brevicompactum	Pbre	F1DBA9
Penicillium herquei	Pher	A0A142C799
Penicillium patulum	Ppat	P22367
Pestalotiopsis fici	Pfic	W3X7U2
Pseudogymnoascus sp.	Pseu	KFY63728
Sordaria macrospora	Smac	A7DWM3
Trichophyton tonsurans	Tton	EGD97507
Usnea longissima	Ulon	AEM75019
Usnea longissima	Ulon	AGI60157.1
Usnea longissima	Ulon	AGI60155
Usnea longissima	Ulon	AGI60156

**Compound/Precursor**	**PKS**
Melledonol (Orselinic Acid)	G8
Patulin	6MSAS
Asparasone (Anthraquinone)	
Unknown	G8
Aflatoxin (Norsolorinic Acid)	G4
Unknown	G8
Terretonin (3,5-dimethylorsellinic acid (DMOA))	G6
Unknown	6MSAS
Oosporein (Orsellinic acid)	G8
Radicicol	G1
Grayanic Acid	G1
Unknown	NR-PKS10
Unknown	NR-PKS12
Usnic Acid	G6
Citrinin	G7
Dothistromin (Norsolorinic acid)	G4
Elsinochromes	
Conidial Yellow Pigment (Naphthopyrone)	G3
Sterigmatocystin	G4
Unknown	G5
Colchin	G6
Citrinin	G7
Anditomin (3,5-dimethylorsellinic acid (DMOA))	G6
Melanin (Dihydroxynaphthalene)	
Zearalenon	G1
Melanin	G2
Hypothemycin	G1
Usnic Acid homolog	G6
Conidial Yellow Pigment (Anthraquinone)	
Citrinin	G7
Cercosporin	
Conidial Pigment Alb1	G3
Endocrocin	G5
Mycophenolic (5-methylorsellinic acid)	G6
Phenalenone	
Patulin	6MSAS
CYP	G2
Unknown	G6
Melanin	G2
Citrinin	G7
Unknown	
Unknown	
Unknown	G6
Usnic Acid	G6
